# Understanding the role of agency in the navigation of regional dementia care and support service pathways

**DOI:** 10.1177/14713012241281620

**Published:** 2024-09-08

**Authors:** Carmela Leone, Rachel Winterton, Marita Chisholm, Irene Blackberry

**Affiliations:** John Richards Centre for Rural Ageing Research, 2080 La Trobe University, Australia; Care Economy Research Institute, 2080 La Trobe University, Australia; John Richards Centre for Rural Ageing Research, 2080 La Trobe University, Australia

**Keywords:** carers, caregivers, dementia care, support services, rural, regional, agency

## Abstract

**Introduction:** Reliable dementia care and support service pathways are essential for timely diagnoses and for reducing the delay in time from diagnosis to care and support. However, carers commonly experience difficulties in finding information about where to go and what to do before and following a dementia diagnosis. In rural and regional areas, accessing dementia care and support services can be especially challenging. This qualitative, narrative inquiry study explores the agency of carers, and people living with dementia, in their navigation of regional dementia care and support service pathways.**Methods:** Semi-structured interviews were conducted with ten carers of people living with dementia from a regional location in Victoria, Australia. Data analysis was guided by the tripartite framework of Giddens’ Theory of Structuration which considered the carers’ intentionality, capacity and power to act in the navigation of their dementia care and support service pathways. **Findings:** Carers had intentionality; however, they did not always have the capacity and power to act. Information played a critical role in facilitating agency. Health literacy was important - as knowledge about where to look for/find information, and knowledge gained through experience, education or learning from others. Where carers encountered barriers, they lacked capacity and power. This occurred where there was an absence of information or knowledge, incorrect information (e.g. misdiagnoses), and where government bodies impeded carers’ efforts.**Conclusions:** Information and knowledge are critical to the progression of dementia care and support service pathways. Health literacy is a significant resource, and carers would benefit from dementia education/training. The agency of carers in navigating their dementia care and support service pathways relies on carers themselves finding information and seeking out knowledge and education. However, GPs, local health providers, and dementia organisations have an important role to play in helping carers to find information towards accessing dementia care and support services.

## Introduction

People living with dementia and carers often navigate pathways to access dementia care and support services together. However, dementia can limit the agency of a person, and decisions are commonly made on their behalf by carers (Bosco et al., 2019), who are largely responsible for pathway navigation. Dementia care and support service pathways are important given that there are currently over 55 million people living with dementia worldwide, with almost 10 million new diagnoses each year (World Health Organization, 2024). Dementia is a syndrome caused by several diseases, which over time, affect the memory, thinking, and ability of an individual to perform daily activities, and which is commonly accompanied by changes in mood, emotional control, behaviour, or motivation (World Health Organization, 2024). The impacts of dementia are physical, psychological, social and economic, and affect not only those diagnosed, but also their carers, families and society at large (World Health Organization, 2024). Informal carers face unique challenges due to the duration and progressive nature of dementia (Engel et al., 2022b), and frequently undertake significant responsibility for the provision of care, often in isolation and without prior training in performing caregiving tasks (Sun et al., 2010). Carers can thereby experience high levels of stress, anxiety, and depression (Jeste et al., 2021). As global rates of dementia increase, so too will the numbers of carers of people living with dementia (Nichols et al., 2022). While the types of support and assistance needed will vary according to personal circumstances, the level of care and support required by people living with dementia will increase with the progression of dementia (World Health Organization, 2024).

Given the prevalence of dementia, and its progressive nature and impact on a person’s abilities and dependencies (World Health Organization, 2024), reliable dementia care and support service pathways that enable the navigation of complex care and support networks are therefore essential, for timely diagnoses and for reducing the delay in time from diagnosis to accessing care and support services (Volpe et al., 2020). Dementia care and support service pathways, however, are largely unexplained to carers, who experience challenges in obtaining information about dementia services (Fitzgerald et al., 2019), and who receive very little information from health care providers (Wiersma et al., 2014), and do not know where to look for information (Samsi & Manthorpe, 2014). In rural and regional areas, further challenges include limitations regarding knowledge about services, access to services, the training of care providers, as well as distance from services, lack of transportation, financial challenges, and stigma (Bayly et al., 2020; Szymczynska et al., 2011). In addition, there is limited access to dementia diagnostic and post-diagnostic services in rural areas (Szymczynska et al., 2011) and limited availability of dementia-related support services, especially respite care (Bayly et al., 2020). There is therefore a need to understand the experiences of carers and people living with dementia in navigating dementia care and support in rural and regional areas. This study addresses an important gap in the dementia literature by focusing on the role of informal carers' agency in navigating the complex territory of dementia care and support services. Drawing on data from an Australian regional setting, this paper interrogates where and how the agency of carers, and persons living with dementia, is facilitated in the navigation of dementia care and support service pathways.

## Background

### Dementia care and support service pathways

The term ‘dementia care pathway’ has been defined in a variety of ways, including as a mechanism for managing uncertainty, as a guide for sequencing care activities, to help consumers understand eligibility criteria for care activities and to guide the self-management of dementia dyads (Samsi & Manthorpe, 2014). A dementia pathway can be diagnostic, experiential, related to clinical services or service management, or it can be a hybrid approach, in which multiple pathways contribute to care and support (Carr & Biggs, 2018). Pathways to dementia care vary across countries, and globally, post-diagnosis pathways tend to be complex (Gauthier et al., 2022). Dementia care pathways are neither straightforward nor linear (Samsi & Manthorpe, 2014), as dementia care and support services may be required some time following a dementia diagnosis, and some carers and people living with dementia may leave and then re-enter pathways.

Studies which map dementia care and support service pathways in community settings have explored the healthcare system pathways of people living with dementia and carers from Australian metropolitan, rural and regional areas (Fitzgerald et al., 2019), and existing care pathways for people living with dementia with fall-related injuries (Wheatley et al., 2017). More broadly, the focus of pathway studies includes post-diagnostic dementia support (Frost et al., 2020), the range of care and services available to persons living with dementia and caregivers (Hallberg et al., 2013), the experiences of carers in their interactions with government, organisations, and informal support or services (Carey et al., 2016), and the dementia services available to people with dementia and their carers prior to diagnosis (Carey et al., 2017).

Several studies map people’s dementia journey experiences, including pathways to seeking a diagnosis (Campbell et al., 2016; Leung et al., 2011; McCleary et al., 2013; Plejert et al., 2017). Studies which map dementia journey experiences commonly focus on gaps and challenges in care provision (Thomas et al., 2023), on the actions, thoughts and feelings of people living with dementia, carers and healthcare professionals (Alzheimer’s Society, 2023), the journey perspectives of people living with dementia, care-partners and service providers (Wiersma et al., 2014), and the care experiences of staff and family members of people living with dementia in care transition (Smith & Phillipson, 2022). Various aspects of the dementia journey have also been a focus of dementia mapping studies, including resource needs (Loup et al., 2023), the drivers and barriers to using dementia-friendly community services (Marshall et al., 2018), and the technology-related tools and services available to people living with dementia (Lorenz et al., 2019). No studies have yet focused on the role of carer agency in navigating dementia care and support services.

### Agency and pathway navigation

According to Giddens (2004, p. 10), “agency refers to doing” and the capability of a subject to produce an effect. Agency is linked to structural influences, which include the availability of material resources for meeting one’s needs, the expectations of others, and internalised views around behaviour (Johnson, 2008). Agency is also linked to external structures, the pressures of which can constrain agency (Stones, 2005). Agency and structure are therefore interdependent, and this study draws on Giddens’ (1984) Theory of Structuration to analyse the interrelations of agency and structure across dementia care and support service pathways. Giddens considered that for an individual to have agency, they “must act intentionally… have the capacity to act on his or her intentions… and have the power to create a new event or to intervene in an existing event” (Varpio et al., 2017, p. 360).

Critics of Giddens’ (1984) Theory of Structuration view his approach as being overly voluntaristic, as overemphasising the agent’s freedom and as being overly optimistic in terms of the competency and skill of the agent (Pérez, 2008). However, this study recognises its usefulness in simplifying the relationship between the agency of individual and the institutions/structures which impact their actions, focusing on only the context within which agency is observed (Lamsal, 2012). This study thereby focuses on the actions of the agent – with consideration for the institutional/social structures which influence their actions – and not on the philosophy or psychology of the action within the context of broader issues. It explores the intentionality, capacity, and power of carers to exercise agency within their dementia care and support service pathways.

The concept of agency has been highlighted in only a few studies relating to carers, where it has been understood to be conferred by the power of knowledge as information, as well as the ability to act on that information (Kinchin & Wilkinson, 2016), and to be constrained due to limited resources and cultural taboos (Daniels-Howell, 2022). Obtaining information and knowledge about dementia is part of health literacy. The role of health literacy has been established in the literature, as enabling carers to make informed decisions which improve the health outcomes of people living with dementia (Engel, Hwang, et al., 2022; Li et al., 2020) and assist in the navigation of dementia care pathways (Giebel et al., 2021; Lorini et al., 2023; Watson et al., 2021). In the agency literature, structural barriers such as prejudices and healthcare infrastructure have been found to limit the agency of patients and carers in their decision-making (Nunes et al., 2019), and cultural as well as structural factors have been found to impact the agency of carers (Cheshire-Allen & Calder, 2021).

As a structural influence, stigma has been found to influence others to avoid carers (Nay et al., 2015), and the relationship between the individual and structural barriers has been recognised, where carers have experienced conflicting medical opinions, misdiagnoses and social stigma in their pathways to and beyond a dementia diagnosis (Brijnath et al., 2021). Outside of the agency literature, there is the recognition that stigma can play a role in the provision of dementia care (Benbow & Jolley, 2012; Hanssen & Tran, 2019). Social stigma can result in the reluctance of carers to seek help and access services (Nguyen & Li, 2020), and to disclose the diagnosis of the person living with dementia, or even their association – which can have significant implications for dementia care (Mukadam & Livingston, 2012). This study contributes to the existing dementia care literature in exploring the agency of carers and structural influences, as they and persons living with dementia navigate dementia care and support service pathways.

## Methods

This study employed a narrative inquiry methodology using semi-structured interview and visual mapping methods. Narrative inquiry involves listening to participants tell their stories through interviews, sometimes with the help of artifacts (Clandinin & Caine, 2013). Visual mapping was used as a co-constructed artifact, to aid in the telling of participant stories. Semi-structured interviews were conducted in 2019 with ten carers of people living with dementia from a large regional catchment area in Victoria, Australia. The region encompasses a large regional service centre, which has a population of more than 120,000 (Australian Bureau of Statistics, 2021) and is located approximately 150 km from the state’s capital city of Melbourne. The region has varying levels of remoteness in relation to health care. Under the Modified Monash Model (MMM) which measures geographical remoteness from the perspective of health care access (Australian Government Department of Health and Aged Care, 2020), the large regional centre is classed as MMM2 (regional centre) and other settlements in the region are classified as MMM4 or MMM5 (with MMM7 being most remote).

Participants were recruited from a larger scoping study which aimed to investigate carer experiences relating to the use of dementia care services within the study area. Purposive sampling was used, and invitations were published in local newspapers, and flyers were sent to dementia care and support service organisations. Interested participants contacted the research team. A short anonymous survey was circulated to carers, which included questions relating to demographics, and the support services and organisations they have used, as well as accessibility information, the factors they found important, and the key challenges they had faced in accessing dementia care services and supports. At the end of the survey, participants were asked if they would like to participate in a follow-up interview. Ten participants registered interest in participating in the interviews. They were phoned to discuss their participation and sent information about the study and a consent form. The consent form was discussed at the interview, prior to obtaining their informed written consent. The interviews were conducted by two researchers – one of whom facilitated the interview with the participant, while the other conducted the mapping/scribing. Participants were notified prior to the interview that two researchers would be present, and the reasons why. The characteristics of interview participants are detailed in Table 1.

**Table 1.** Carer characteristics.

Carer ID	Carer age	Carer sex	Relationship to person living with dementia	Years since diagnosis	Years Caring	Age of person living with dementia	Sex of person living with dementia	Living situation
Carer 1	71	Female	Wife	2.5–3	2	69	Male	At home
Carer 2	73	Female	Friend	8	5	80	Male	RACF
Carer 3	59	Female	Daughter	5	20	83	Female	At home
Carer 4	68	Female	Wife	8	10	72	Male	RACF
Carer 5	77	Female	Wife	3	3	84	Male	At home
Carer 6	73	Female	Wife	1	1	77	Male	At home
Carer 7	58	Female	Daughter	11	4	87	Female	RACF
Carer 8	51	Female	Daughter	8	8	81	Female	RACF
Carer 9	61	Female	Wife	6	5	68	Male	RACF
Carer 10	81	Female	Wife	11	10	91	Male	RACF

One participant was from a non-English speaking background (Carer 3) but had a high level of proficiency in English. All of the carer participants were female, with a mean age of 67 years, and most were spousal carers, with just over half of the persons living with dementia in a Residential Aged Care Facility (RACF) at the time of the interviews. The mean years that carers had been undertaking care responsibilities was 6.8 years, and the mean age of the persons living with dementia was 79 years. While every one of the participant stories were unique, data saturation was deemed to have been reached at ten interviews, with each story sharing similar elements in terms of seeking a diagnosis and accessing support services, even though their outcomes may have differed, or they may have experienced delays.

Ethical approval was obtained from the La Trobe University Human Research Ethics Committee (HEC18377) in 2018. Visual mapping techniques coupled with semi-structured interview methodologies were employed to explore how carers navigate dementia care and support services, and their experiences of service access. Visual mapping is a method of presenting and organising information in a visual format, and includes mind maps, concept maps and conceptual diagrams (Choudhari et al., 2021). It is a participatory, qualitative research method that is beneficial in prompting more in-depth responses from participants, and leads to better recall, organisation and framing of their past experiences (Bahn & Weatherill, 2012; Wheeldon, 2011). The visual mapping method detailed the services that carers accessed and the order in which those services were accessed, effectively mapping their dementia care support journey. The interview schedule consisted of three sets of questions. The first set of questions aimed to elicit visual mapping, through understanding when carers accessed services, from where they accessed them, how they found out about them and any issues that were encountered. The responses informed the first map. The second set of questions sought to capture the experiences and stories of the carers, including why they accessed the services, what helped or hindered them and how satisfied they were with the services. These responses provided more information to refine the map. The last set of questions collected demographic information. Because carer narratives were not always linear, the maps were a visual representation of the dementia journey, which included the different stages of the pathway trajectory.

The mean time of the interviews was one hour and 15 minutes and were conducted with two researchers present. The first researcher acted as the interviewer while the second researcher simultaneously visually recorded the information to create a user-generated, directional map of how carers had engaged with services. This was amended and reworked with the carers and was used as a prompt to elicit information in relation to accessing services, the reasons why they selected services, barriers to accessing services, any factors that facilitated access and their level of satisfaction. Because narrative inquiry involves the interpretation of the narrator’s experiences by both the narrator, through telling their story in their way, and the interviewer, who interprets the meaning of the narrator’s story, the process of meaning-making is a joint production (Hiles & Cermák, 2008). The process of visual mapping also is a joint meaning-making process. Narrative inquiry is a relational research methodology, and ethical issues are therefore central to inquiry (Clandinin & Caine, 2013), and both researchers paid attention to possible ethical tensions in their relationships with participants, and represented to the best of their ability, the lived and narrated experiences of participants. Interviews were audio recorded and transcribed and entered into a qualitative analysis program (NVivo).

Except for one interview (Carer 1), all carers were interviewed without persons living with dementia present. In this interview, the carer elected to have the person living with dementia with her, even though it was optional. The researchers recognised that it would have been unethical to exclude the person living with dementia and assessed his demeanour and willingness to consent throughout the interview. In all other interviews, carers spoke about and on behalf of the persons living with dementia. Most of the discussions in the interviews focused on post-diagnosis pathways, and the agency of the carers is therefore the focus of the findings. Where carers relayed accounts of seeking a diagnosis, it was not always clear whether the persons living with dementia exercised agency in that decision. However, it is not uncommon for carers to increasingly take responsibility for decision-making in their relationship with a person living with dementia (Samsi & Manthorpe, 2013). Furthermore, persons living with dementia are often self-aware and have some notion that something unusual is happening, well before a diagnosis (Campbell et al., 2016).

## Analysis

Data was coded inductively in NVivo in the first instance, to identify the factors which impact the carer’s navigation of the dementia care and support service pathways. Data was then coded deductively using Giddens’ (1984) Theory of Structuration and the three requirements for agency, being intentionality, capacity, and power, to explore agency at different stages in carer trajectories. Inductive analysis of the transcripts highlighted several junctures along carer pathways where progress was facilitated or challenged. At each juncture, analysis determined whether carers, and persons living with dementia where applicable, had the intentionality, the capacity and/or the power to act, and to therefore be agentic during their navigation. In the analysis, intentionality refers to the intentional actions of a subject – an intentional act being “an act which its perpetrator knows, or believes, will have a particular quality or outcome and where such knowledge is utilized by the author of the act to achieve this quality or outcome” (Giddens, 2004, p. 10). Capacity refers to the ability of a subject to achieve their desired outcomes (Giddens, 1999), and power, to the capability of a subject to make a difference, including the ability to deploy causal power, and to influence powers deployed by others (Giddens, 2004). The institutional/social structures which influenced their pathways were also explored.

## Findings

**Figure 1.**
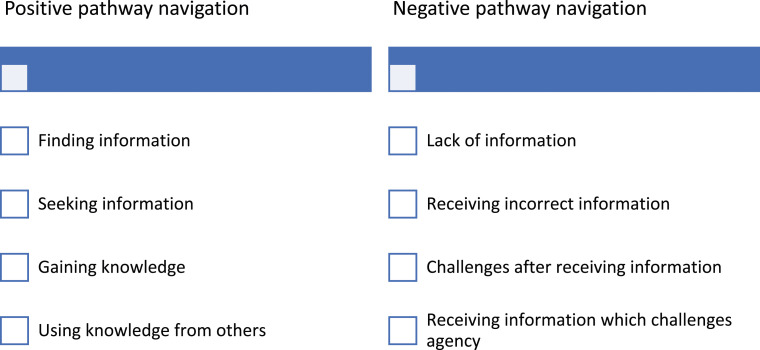
Pathway Navigation.

Most of the discussions in the interviews focused on post-diagnosis pathways, and the agency of the carers is therefore the focus of the findings. Within narrative inquiry, participants look inward, outward, forward and backward in telling their stories (Clandinin & Connelly, 2000); in looking backward to tell their stories, carers tell their stories from a position of having acquired knowledge and experience. Data analysis revealed themes of positive and negative pathway navigation. Where the trajectories of carers and persons living with dementia progressed without major disruptions, and where carers had the intentionality, capacity, and power to exercise agency, pathway navigation was positive. Where trajectories were disrupted or halted, and where carers had the intentionality but lacked the capacity and the power to exercise agency, experiences of pathway navigation were negative. Sub-themes relating to information and knowledge were also identified (Figure 1).

### Positive pathway navigation

Across all pathways, carers can be seen to have acted with intentionality. While carers may have been forced in a particular direction, when they acted, they did so with the intention of producing certain results, such as obtaining a diagnosis and finding information or care.

#### Finding information

Information played a critical role in facilitating agency, as it provided a signpost to guide carers on their pathways. Through the provision of instruction, guidance, direction and/or details about where to go and/or what to do next, information facilitated the agency of carers to make decisions and act. The important role of information highlighted the relationship between health literacy and the capacity to take action (Rademakers & Heijmans, 2018). Some carers who lacked information found ways of moving forward, nonetheless. Carer 4 lacked capacity and power, as she was not provided with information by her GP following the diagnosis. It was some time after that she found information about a carer’s group in the local paper, which helped to progress her pathway:

I reckon it was about 18 months, two years, and I just really, I was never pointed anywhere else, so I didn't know to do anything else, but I had read, in the local paper, where they had started a group, based in (local town), for carers of people with dementia, so I picked up the phone… I started going to the group. (Carer 4)

The information provided her with direction, and therefore with the capacity to seek support. Similarly, information contained in a letter about a dementia lecture was critical to the ability of Carer 1 to exercise agency in finding information about dementia, as she states that, *“we wouldn’t have known anyway – otherwise”* (Carer 1). Receipt of another letter led to the long-term membership and participation of both her and her husband in a local group: *“We received a letter that told us about the Style Café, and what it was about. Yes, we’ve been members of that for almost two years”* (Carer 1). At both junctures, information provided the carer with guidance and facilitated her agency to progress her pathway.

#### Seeking information

Positive pathway navigation was also observed where carers who lacked information took control and persisted in seeking direction, often in the face of challenges. In these instances, there were no signposts; the carers’ capacity depended on their ability to discover information, and their power relied on self-directed action and/or proactivity. In the pathway of Carer 2, efforts to improve and change the trajectory of care for her friend were met with obstacles, such as delays in finding doctors and specialists, and regularly changing case plans. These structural influences acted as barriers. Nonetheless, Carer 2 took control by persistently pursuing information: “*I’ve had to ask for everything… For me, it’s just been a huge struggle all the way along… Just hate to think what it would be like for people that have got no idea. Kind of almost need some sort of advocacy to walk you through it.”* (Carer 2). Carer 2’s reference to advocacy reflects the need for support and guidance where information is difficult to find, and the potential powerlessness of carers who lack direction. That Carer 2 had some idea of who and/or what questions to ask to progress her pathway may reflect her experience, as in her interview she mentioned her background in nursing and case management. Carer 2’s knowledge of and/or experience with the medical system empowered her.

For Carer 4, a lack of information about potential respite activities for her husband contributed to a lack of direction. She had no guidance and there were no signposts, and she therefore lacked the capacity and power to act. However, she empowered herself by seeking information:

That was the only way. It was only that I thought, well, where would there be group activities? Well, try the Community Health Centre… not once did a GP say to me, maybe you should investigate this. I'd not had anyone in the family with dementia. I knew nothing about dementia… It was if I didn’t seek something out, there was nothing else. (Carer 4)

Carer 4 acknowledged that information and guidance could be found in having some knowledge of dementia, such as through experience with a family member.

#### Gaining knowledge

Knowledge through education and learning about dementia also facilitated agency for some carers, as it empowered them and gave them confidence to provide care. For Carer 3, training sessions and regular activities run by a carer support group and Alzheimer’s (now Dementia) Australia provided her with guidance to better support her mother. She gained knowledge through regularly meeting with other carers:

Basically, I was playing by ear. Learning from listening to those people. I’ve joined a couple of activities that they’re running… We meet, and have lunch, or tea, or whatever, in various locations. It’s kind of - you’re starting to know people, and telling your story, they tell their story, and you learn from that quite a lot… I feel more confident that I can manage any situation that might occur. (Carer 3)

Knowledge from a financial planner about care options facilitated agency for Carer 10:

That was the most helpful thing that had ever happened. She walked me through the whole system... She came with me on the day that I signed the contracts… because I had no idea, and I had all the wrong ideas about how to finance this. She gave me a good option that has been manageable. So, that was sort of key to negotiating the system. (Carer 10)

With the knowledge provided by the planner – who was recommended by someone at a care facility – Carer 10 had the capacity to find affordable care for her husband.

#### Using knowledge from others

Where some carers lacked power due to roadblocks, interventions from representatives of a local agency – who had knowledge of the system, and status within the institutional landscape – facilitated their agency to progress their pathways. Carer 2 experienced delays in receiving an assessment to obtain respite services and permanent care for her friend. Following regular phone calls over a period of five months to the service provider, a person from a local care agency intervened, and the carer received an assessment from the Aged Care Assessment Service (ACAS): “*They put a bit of pressure on ACAS too, to get out and get it done. It was just the last minute we got on”* (Carer 2). Similarly for Carer 3, after receiving contradictory information from a local agency and My Aged Care about eligibility for support, a representative from the local agency intervened:

You get fed up, and I don’t know if this is the point [laughs]. To get fed up, and not to have services provided by My Age, or God knows. It’s ridiculous… one lady from (local care provider), she contacted My Aged [Care], and they’ve sorted out for that, because I couldn’t…I couldn’t sort it myself. (Carer 3)

In both pathways, the local agency acted as a structural influence to remove the roadblocks created by larger institutional structures (ACAS and My Aged Care).

### Negative pathway navigation

Data indicates that carers had the intentionality to act across all negative pathways, however, as they lacked information such as instructions, directions, or knowledge to inform their navigation, they lacked capacity. Without information or knowledge about where to go or what to do, carers also lacked power.

#### Lack of information

In negative pathway navigation, there were no signposts, and carers lacked capacity and power at junctures where they did not receive information about the steps to take following a diagnosis. Carer 1 was not provided with instructions following a dementia diagnosis: *“We weren’t told what to do. Just received a diagnosis, and then thought, what do we do?”* (Carer 1). Similarly, Carer 4 was unable to move forward following a diagnosis without direction and guidance: *“I think that was probably the difficult part, because once we were given that diagnosis… I really would have loved it if they had have just referred me somewhere”* (Carer 4). For both carers, navigation of their pathways came to a standstill at these junctures. Carer 3 – whose mother was also living with another significant illness – was directed by the Memory Clinic to consult her mother’s case manager. However, a lack of direction and instruction meant that she was unable to progress: *“I did not get too much help from them. So, it was kind of, what am I doing now? I did not know what to do, so, basically, for two years… I wasn’t able to do anything for her… just taking care of her, managing on my own*” (Carer 3). In this respect, Carer 3 was navigating two pathways – with the dementia services and support pathway taking a lower priority – and she was unable to progress either of them due to a lack of information. For Carer 8, it was following an education session that she found herself without direction, and therefore without the capacity and power to act:

They offered an education thing after diagnosis. That came within a couple of weeks I think, which was good… it was a big group of people, and they just gave out the facts, and there you go sort of thing. From that point, that was it. It was like, get on with your life, whatever, that was the end of it. (Carer 8)

In each of these situations, structural forces – as institutions which failed to supply the carers with information – challenged the capacity and power of the carers to exercise agency.

#### Receiving incorrect information

Some carers and persons living with dementia received incorrect information regarding the diagnosis, and the persons living with dementia were unable to receive appropriate care and support in a timely manner. In these pathways, structural forces – as health professionals – disrupted and delayed pathways. While carers and persons living with dementia had intentionality in seeking a diagnosis, in receiving a misdiagnosis, they lacked the capacity and power to find appropriate care and support. In the pathway of Carer 4, her husband was diagnosed with stress by several GPs, and she thereby sought relevant treatments:

Then I decided to pursue other forms, like went to Chinese medicine, acupuncture, thinking, well, if it was just stress, just get everything - the body balanced, then - and he'd started losing weight, as well, and his health did pick up a lot. It was finally, one of the guys that we were going to for that, said, look, I'd like you to go to a GP - and this time just ask for a referral to a neurologist. (Carer 4)

Similarly, in the pathway of Carer 1, when her husband began experiencing memory issues, he was directed to a specialist by his GP who diagnosed a mini stroke: *“But he still hadn’t been diagnosed with dementia… he had a PET scan done and a CT scan… They noticed - they thought that he’d had a stroke, a mini-stroke… They still weren’t sure what it was, but that was the first diagnosis, that he’d probably just had a mini stroke”* (Carer 1). When the carer and her husband moved house and found another GP, a dementia diagnosis was finally given, and navigation towards receiving dementia support and services began.

#### Challenges after receiving information

The capacity and power of carers to exercise agency fluctuated over the course of their trajectories, as they received information which facilitated progress at certain junctures, and then met challenges which disrupted/halted their progress at others. Carer 3 was empowered to find dementia care and support for her mother when she found information about care packages: “*When I went to that session - training session, run by Dementia Australia and Carer Support Group, there were - the people there, they had the package. What is this?*” (Carer 3). When Carer 3 approached My Aged Care, she encountered roadblocks in navigating the system: *“And they said, no, she’s not entitled, because such and such. I’m getting that message from My Aged [Care]. Going to the (agency), to ACAS, again, and ask - no, no, she’s got - she’s - and it’s kind of - I said, I’m giving up”* (Carer 3). The carer was further disempowered by structural obstacles:

I’m still trying to find out what Mum is entitled to. Because My Aged Care, they don’t have her in the system. I cannot access the system. I don’t know anything, so I have no idea. I’m waiting for other people to call me and to tell me… I can call them, but every single time you have to wait, and it’s kind of… you reach to frustration at the end of the day. (Carer 3)

At this juncture, Carer 3 lacked both capacity and power, as she could not obtain information regarding care package entitlements, and in being reliant on others, she was powerless. Institutional processes and structural forces prevented her from making progress.

#### Information which challenges agency

Legal requirements were a structural influence in most pathways, where carers received a letter from VicRoads (the state licencing authority) requesting that the person living with dementia undertake a compulsory driving assessment. For Carer 1, the assessment raised concerns about mobility and access, as she did not drive and was dependent on her husband: *“What will happen in the future, more than anything. Because if [husband] loses his licence, we have to leave here, we can’t stay here. Because there’s no public transport. But we don’t want to leave here”* (Carer 1). The agency of both the carer and her husband would be impacted by the revocation of her husband’s driver’s licence. While a diagnosis does not mean that a person living with dementia needs to immediately surrender his/her driver’s license, due to the gradual decline in their cognitive and physical abilities, they will at some point need to stop driving (Dementia Australia, 2022). Following Carer 1’s statement, the facilitator asked her if knowing about available transport options would help to support her and her husband. Both she and her husband agreed that it would help them to plan the next stage of their dementia journey. In this respect, information about transport alternatives would have provided them both with the potential to exercise agency in addressing their mobility needs.

## Discussion

Through the deconstruction of agency and a focus on the role of structural influences, this study contributes new knowledge about how carers developed capacity and empowered themselves to exercise agency in the face of structural barriers. This study thereby provides new insight into how carers can better support themselves in navigating dementia care and support services, and how various institutional/social structures can better provide carers with support. The findings complement existing dementia pathways research which shows that care service pathways are largely unexplained to carers, that information about dementia services is difficult to obtain (Fitzgerald et al., 2019), and that few carers know where to look for information (Samsi & Manthorpe, 2014). It also complements mapping research which indicates that carers receive very little information from health care providers following a diagnosis (Wiersma et al., 2014). This study, however, demonstrates where and how carers were able to progress their dementia care and support service pathways, by locating the information they needed by themselves or with the support of external parties.

### Health literacy

The importance of finding/receiving information for the progression of dementia care and support service pathways demonstrates that health literacy is a prerequisite to taking action (Rademakers & Heijmans, 2018), and furthermore, that health literacy can empower individuals (Crondahl & Eklund Karlsson, 2016). In the field of health literacy, there are concerns that the responsibility for finding health-related information is shifting from society to the individual (Razum et al., 2016). This may be seen in the pathways where carers and/or persons living with dementia were not provided with information from their GP about what to do or where to go following a diagnosis. Health literacy includes the “ability of individuals to gain access to… information in ways which promote and maintain good health” (Nutbeam, 2008, p. 2074). Relatedly, health information literacy expands on this to include knowing how and where to find information about health (Eriksson-Backa et al., 2012). Both concepts are reflected in the findings, with some carers demonstrating health information literacy in locating information, despite not receiving support from their GP or others. While Carer 3 had a high proficiency in English, language barriers and health literacy can pose a challenge for carers from non-English speaking backgrounds – not only in terms of understanding information, but also with respect to interpreting important technical information which could impact their relative’s access to dementia services (Gilbert et al., 2022).

Carers who found/received information in local newspaper ads and in letters from a local health service/memory clinic were able to progress their pathways following a diagnosis. In these instances, newspaper media and health services, as institutional structures, facilitated carers’ agency, and provided direction which resulted in their participation in carer groups and dementia education. Notably, at these junctures, the carers were passive actors, and their trajectories may have differed had they not read the paper/received a letter. This highlights the importance of the media to the dissemination of information about dementia services to carers, and the need for health providers to communicate with carers following a diagnosis (Lai & Chung, 2007). Memory clinics and GPs in particular, have an important role to play in providing information about local services (Abley et al., 2013). Understanding who disseminates information and where one can find it can empower carers to progress their pathways. If carers know to look in their local papers or call their local health service/memory clinic for information, they can be actively agentic in their pathways.

### Resources

Carers who did not find or receive information from the paper or service providers developed capacity in their pathways by proactively seeking information for themselves, by asking (sometimes persistently) those they believed would be most likely to have the information i.e., service providers and community centres. While lacking information and therefore capacity, they had the intention of finding the information they needed, and they had power – as time and resources they could draw on. This is important, as while seeking out information facilitates agency for carers, many do not have the time and chasing up information can be stressful (MacLeod et al., 2017). In these cases, advocacy from other institutional structures such as local service providers, can be an important resource. This was evidenced in pathways where ACAS and My Aged Care had failed to supply carers with the information they needed to act, and a local service provider intervened to exert their institutional influence, to progress the delayed assessments/information. Carers were then empowered to progress their pathways. This finding suggests that carers may be able to draw on local service providers as a resource, to intervene in delayed institutional processes.

In addition, as suggested by Carer 4, knowledge and experience with dementia can be a useful resource. Certainly, dementia knowledge provided through training sessions and carer groups facilitated capacity for some carers. This is supported by research which shows that dementia training to carers plays an important role in dementia care (Sutcliffe et al., 2015), and that education, as well as information, is critical to the navigation of dementia pathways (Wiersma et al., 2014). The direction and guidance provided to carers by dementia education helped them to achieve better outcomes for persons living with dementia and empowered them to manage and improve their situations. Institutional structures such as Alzheimer’s Australia (now Dementia Australia), and carer groups – which act as social structures for carers, through shared identity and experiences – facilitated the transference of knowledge, through training and group activities. This suggests that dementia training should be included in dementia care and support service pathways, either through direction from GPs following a diagnosis or communications from dementia training organisations. Promotion of dementia education is critical; if carers know that it exists, they can then seek out local training that suits their needs.

### Barriers

Carers were not always able to develop capacity as some were powerless at certain junctures. Where carers experienced negative pathway navigation, institutional structures created roadblocks or barriers, causing trajectories to come to a standstill. Misdiagnoses from GPs caused delays and diverted carer resources elsewhere, as carers sought support and treatment for other conditions. Correct diagnoses were eventually provided following a redirection to a different GP or a specialist, with delays costing carers and the persons living with dementia up to two years in lost time. Time is very important in a dementia journey, not only in terms of obtaining a timely diagnosis, but because dementia can progress quickly, and dramatic life changes can leave little time for the carer to seek information (Geoghegan, 2022). Research shows that the reluctance or failure of a GP to provide a ‘timely’ dementia diagnosis can reflect a range of judgements, which are balanced and negotiated in varying contexts, with many prioritising the right time to diagnose over an early diagnosis (Dhedhi et al., 2014). Nonetheless, this finding suggests there is value in carers educating themselves, to improve their awareness of dementia so that an early diagnosis can be made and subsequent access to support can be found (Sutcliffe et al., 2015). GPs can also be more aware of the factors which contribute to a misdiagnosis, including the failure to adopt standardised diagnostic criteria, and the presence of mood disorder symptoms, so they can protect patients from an incorrect dementia diagnosis (Howard & Schott, 2021).

The Australian Government’s aged care system also caused delays. As a powerful institutional structure, My Aged Care created roadblocks which disempowered and immobilised carers. Similar systemic issues with navigating aged care services have been observed in other countries such as England (Peel & Harding, 2014) and Canada (Funk, 2019). While institutional processes can be disempowering to carers, accessing My Aged Care as soon as possible following a diagnosis can empower carers and help to avoid delays in accessing care and support (Alzheimer’s WA, 2023). VicRoads was also an institutional influence, in the case of Carer 1, where her husband was the only driver, and where the loss of a driver’s license would limit mobility and access for both her and her husband. This pathway highlights an interdependency that can be found within carer-person living with dementia partnerships (Keyes et al., 2019). As indicated by Carer 1, knowledge about travel options can assist with journey planning. Travel options include taxi subsidies and community transport (Forward with dementia, 2021); public transport infrastructure and government programs for subsidised taxi travel are therefore institutional structures which can influence outcomes for carers and persons living with dementia. Knowledge about the available travel options can empower carers to exercise agency, and information could be disseminated early in the dementia journey, by GPs following a diagnosis. This would also prepare persons living with dementia and carers for the inevitability of license revocation.

## Conclusion

Resources to help carers navigate dementia care and support service pathways are available online (NHS England, 2022; The Good Care Group, 2022), including a web-based repository of dementia-related information, tools, service directories and resources to help carers navigate their journeys (Centre for eResearch and Digital Innovation, 2024). These sites recognise that information is critical for carers to progress their dementia care and support service pathways. Health literacy and the ability of a carer to locate information, or to garner the support of external parties to locate information is a significant resource, particularly for carers who lack knowledge of or experience with medical or governmental systems. Knowledge and education are also crucial, and carers would benefit from dementia training following a diagnosis. The responsibility for being informed or educated is not up to carers alone however, and direct contact from dementia training organisations, or the promotion of dementia education would help carers to find local training that suits their needs. GPs also play a role, in being aware of the factors which contribute to a misdiagnosis, in directing carers to support services and by providing information about taxi subsidies and community transport. Undoubtedly, the agency of carers in navigating their dementia care and support service pathways relies on several organisations and groups working together, so that they can find and secure the support they need to care for persons living with dementia.

In closing, some limitations of the research should be noted. Because the interviews and visual mapping were conducted some years after the events took place (for two carers, it was over a decade since diagnosis of the person living with dementia), participants’ ability to recall their stories accurately is recognised as a limitation of this study. Furthermore, those who care for people living with dementia do not always identify as carers. The authors also recognise that the study attracted female carers only, with the literacy skills, ability and capacity to participate in research, and that their stories do not reflect those of all carers. Given that participants were primarily from an English-speaking background, the experiences of carers who have low levels of proficiency in English, and different cultural norms and experiences relating to dementia or service navigation, must be investigated more closely. Lastly, carer experiences of accessing services in a regional community are not reflective of the experiences of carers in metropolitan areas or more remote localities and should be investigated separately. Nevertheless, this small study conducted in one Australian state provides some critical insight into factors that both support and hinder carer agency relating to dementia service navigation, which is important in developing targeted strategies to support access to services.
